# Supplementary material for: Integrative Analysis of Intrahepatic Cholangiocarcinoma Subtypes for Improved Patient Stratification: Clinical, Pathological, and Radiological Considerations
Source: Cancers (Basel). 2022 Jun 28;14(13):3156. doi: 10.3390/cancers14133156 (PMC9264781; doi:10.3390/cancers14133156)
Supplement: Supplementary file 1 [file cancers-14-03156-s001.zip › cancers-1779888-supplementary.pdf]

cancers MDPI

Table S1. List of antibodies.

Antibody	Supplier	Clone	Type	Dilution	Antigen Retrieval
Anti-CK7	Dako	OV-TL	Mouse monoclonal	Ready to use	Tris/EDTA buffer, pH 9
Anti-CK20	Dako	K2 20.8	Mouse monoclonal	Ready to use	Tris/EDTA buffer, pH 9
Anti-Ca19-9	Dako	1116-NS-19-9	Mouse monoclonal	Ready to use	Tris/EDTA buffer, pH 9
Anti-EMA	Dako	E29	Mouse monoclonal	Ready to use	Tris/EDTA buffer, pH 9
Anti-S100	Dako	IR504	Rabbit polyclonal	Ready to use	Tris/EDTA buffer, pH 9
Anti-CD56	Dako	123C3	Mouse monoclonal	Ready to use	Tris/EDTA buffer, pH 9
Anti-N-cadherin	Biosciences	Clone32	Mouse monoclonal	1:500	Tris/EDTA buffer, pH 9
Anti-CRP	Abcam	Ab32412	Rabbit monoclonal	1:1000	Tris/EDTA buffer, pH 9
